# An Integrated Conceptual Model to Understand Suicidality among Queer Youth to Inform Suicide Prevention

**DOI:** 10.3390/soc12060170

**Published:** 2022-11-22

**Authors:** Denise Yookong Williams, William J. Hall, Hayden C. Dawes, Cynthia Fraga Rizo, Jeremy T. Goldbach

**Affiliations:** 1 School of Social Work, University of North Carolina at Chapel Hill, Chapel Hill, NC 27516, USA; 2 Brown School, Washington University in St. Louis, St. Louis, MI 63130, USA

**Keywords:** queer, LGBTQ, youth, adolescence, suicide, mental health, intersectionality, minority stress, prevention, theory

## Abstract

In this article, we apply and combine elements from four theoretical frameworks (i.e., Minority Stress Theory, Person-in-Environment and Risk and Resilience Framework, Interpersonal-Psychological Theory of Suicide, and Intersectionality) to explain the problem of queer youth suicide through our integrated conceptual model, Queer Prevention of Youth Suicidality Model (Queer-PRYSM). The need for this conceptual model is based on the current state of the literature, including mixed empirical findings on factors related to queer youth suicidality, no scholarly consensus on specific contributing factors regarding high rates of suicidality among queer youth (including queer youth subgroups), and the absence of a unifying theory to explain the queer youth suicide risk. To address these limitations in theory, evidence, and scholarship explaining suicidality among queer youth we present our integrated model with growing, current, relevant research with queer youth. Queer-PRYSM includes minority stressors specific to queer youth, mental health problems, interpersonal-psychological factors, socioecological factors (i.e., family, school, peers, and community), and intersectionality concepts. Queer-PRYSM is essential to understanding the relationship of distal and proximal risk and protective factors in queer youth suicide and developing evidence-informed suicide preventive interventions that can be incorporated into practice, policy, and system structures.

## 1. An Introduction to Youth Suicidality in the United States

Adolescent suicide is a pressing public health concern and grand challenge for society [1,2]. Among youth ages 10 to 24 years, suicide consistently ranks as a leading cause of death [3]. In 2019, in the United States alone, suicides accounted for at least 22% of all deaths in youth ages 15 to 19 years [4], with estimated national costs of $70 billion [3]. Despite a slight drop in 2019 [5], youth suicide rates have continued to climb in the United States in the 21st century [6]. Moreover, suicide risk and rates are exacerbated among youth with historically marginalized identities based on sexual orientation, gender identity, disability, class, race, and other characteristics [6-10].

## 2. Queer Youth Suicidality

In the United States, an estimated 1,994,000 youth, or roughly 10% of the adolescent population ages 13 to 17 years, self-identify as queer [11]. We will broadly use the term “queer” throughout our manuscript as an efficient and inclusive umbrella term for those with a non-heterosexual and/or non-cisgender identity (e.g., lesbian, gay, bisexual, pansexual, transgender, non-binary+). However, it is important to note that sexual orientation and gender identity are distinct aspects of an individual and should not be conflated. As such, when appropriate, sexual orientation and gender identity are explicitly and distinctly explored more in-depth in some sections of our manuscript (i.e., intersectionality; when discussing mental health disparities among subgroups of queer identities) and referred to separately.

As a socially marginalized group, queer youth report disproportionate negative suicidality outcomes compared to their heterosexual and cisgender counterparts [12,13]. The 2019 Youth Risk Behavior Survey (YRBS) shows that 44–47% of queer youth (i.e., gay, lesbian, or bisexual youth per their collected demographics) seriously considered suicide in the past year, compared to 15% of their heterosexual/cisgender peers [14,15]. Additionally, 23–29% of queer youth attempted suicide in the past year, compared to 6% of heterosexual/cisgender youth [14,15]. Though numbers vary depending on specific identities (e.g., transgender males and nonbinary youth report higher suicide attempt rates), some research even suggests that between 30–51% of transgender teenagers have previously attempted suicide in their lifetimes [13]. Notably, as sexual orientation and gender identity are not consistently recorded in death investigator reports, accurate queer youth suicide mortality rates remain unavailable [16]. That said, compared to heterosexual and cisgender youth, queer youth report more concerning trajectories of both suicidal ideation and suicidal behaviors over time, with a younger age of onset of suicidality, a disproportionately higher number of suicide attempts, and a stronger desire to die [16]. However, these percentages may not accurately reflect the current state of suicide-related behaviors considering that under-reporting is common in suicide risk screening and research [17,18].

Suicide-related disparities also exist at the intersection of race and sexual orientation, in addition to other identities. Roughly 56% of multiracial, 57% of Asian, 52% of White, 39% of Latine, 35% of Black or African American, and 31% of Indigenous queer youth seriously considered suicide [14,19]. With sexual orientation and gender marker data collection largely lagging behind other demographic characteristics, few nationally representative studies have explored these intersections. Additionally, it is important to note that, when considering newly emerging sexual and gender identities, as well as multiply marginalized identities, small sample sizes in particular demographics may make research difficult even in large, nationally representative survey studies [20]. Though evidence is emerging regarding suicidal ideation and suicide risk rates among queer youth with diverse racial/ethnic identities, for example, among other intersecting identities, such as queer youth with disabilities, the evidence is even more limited. Nevertheless, emerging evidence shows that queer youth with disabilities are at a higher risk of experiencing suicidal ideation compared to both heterosexual youth without disabilities and queer youth without disabilities [21]. In addition to existing suicide risk disparities among youth with multiply marginalized identities, queer youth have significant barriers in accessing mental health treatments and supports to address their needs [22,23], compounding risk. Taken together, these findings illustrate the importance of intersectional factors and in using an intersectional framework to examine queer youth suicide risk.

Suicide is influenced by social context in complex ways. Starting with a sociological perspective and continuing to include social determinants of health, broadly, and structural racism and inequalities [7,24], specifically, suicide is a complex and multifaceted problem that can be preventable. This problem is undergirded by social marginalization as evidenced by the high rates of suicidality among queer youth and intersectional disparities (e.g., queer identity and race/ethnicity), representing a serious public health problem and grand challenge for society [1,25].

## 3. Need for a Conceptual Model on Suicidality among Queer Youth

An integrated conceptual model is needed to identify mechanisms contributing to suicidality among queer youth which focuses on the interplay of relationships between both proximal (i.e., individual) and distal risk and protective factors (e.g., systemic racism, community influences). A model offers a way to capture the various systems and intricacies related to this particular issue, to inform problem conceptualization, research, and potential interventions at various levels (e.g., individual, systemic). This is especially important for queer youth. Though exploratory work has been conducted with queer youth, there are mixed findings on specific contributing factors toward disparities in suicide risk among queer youth subgroups (e.g., bisexual versus gay or lesbian; transgender individuals and/or gender non-conforming versus cisgender; [16,26]), making it difficult to develop and research appropriate prevention interventions for this unique population. Compounding this lack of scholarly agreement on the relationships of suicide risk factors affecting queer youth is the dearth of research focused specifically on transgender youth and subgroups (e.g., considerations regarding assigned sex at birth, transgender females, transgender males, or gender non-conforming individuals), though some scholars are attempting to address this gap ; [13,27,28]. There is also extensive variability among correlates of suicidal ideation and suicidal behaviors within queer adolescent populations in particular—extant research oversimplifies the relationships for factors associated with queer youth suicidality (e.g., depression, suicidal ideation, and suicidal behaviors), focusing on positive and negative correlates/predictors, rather than complex mechanisms and pathways [29,30]. There is a dearth of theory, evidence, and scholarship explaining suicidality for particular population groups in specific developmental phases, such as queer youth, and regarding protective factors for queer youth suicidality [26,31]. While some leading researchers in the field of youth suicide are advancing more comprehensive understandings of suicide risk and suicide prevention in youth with historically marginalized identities (e.g., youth of color [7]) and considering macro-level influences on individual-level risk factors (e.g., [10]), to-date, to our knowledge, there is no existing model that comprehensively helps to explain queer youth suicide.

The present paper proposes an integrated conceptual model to understand suicidality among queer youth in an effort to guide the development of appropriate preventive interventions. The Queer Prevention of Youth Suicidality Model (Queer-PRYSM) is essential to understanding the relationship of distal and proximal risk and protective factors in queer youth suicide and developing evidence-informed suicide preventive interventions that can be incorporated into practice, policy, and system structures. Considering that “… theoretically pertinent constructs that are relevant to our understanding of suicidal thoughts or behaviors among LGBTQ youth are largely absent” [26] (p. 31), our conceptual model draws on multiple theories and frameworks based in psychology, social work, sociology, public health, and related fields to provide a comprehensive, multidisciplinary conceptualization of suicidality among queer youth to address this area of need and understanding. The model integrates four conceptual frameworks or theories: Minority Stress Theory [32,33], Person-in-Environment and Risk and Resilience Framework [34], Interpersonal-Psychological Theory of Suicide [35], and Intersectionality [36]. Below, we briefly describe each of these theories/frameworks. Then, we describe the conceptual model to explain suicidality among queer youth incorporating the extant research and theoretical principles. Finally, we discuss directions for future research and potential application of the model for practice, policy, and prevention.

## 4. Theories and Conceptual Frameworks to Understand Suicidality among Queer Youth

Theories and frameworks relevant to understanding suicidality among queer youth are outlined below, providing basic concepts and definitions included in our integrated model.

### 4.1. Minority Stress Theory (MST)

MST [32,33] is the leading theory in research that seeks to understand mental health problems among queer people. MST postulates that sexual and gender minorities experience unique, chronic, and socially based stressors because of their stigmatized and minoritized status [33]. Discrimination and stress may stem from identities related to race, sexual orientation, and gender identity [33,37]. Minority stressors, including *distal stressors* (e.g., homophobic and/or transphobic abuse or victimization), and *proximal stressors* (e.g., fear of discrimination, rejection, and internalized stigma), are additive to typical life stressors faced by the general population (e.g., death of a loved one, job loss [33]). MST posits that stressors manifest across a continuum, from external to internal processes, each with significant impacts on mental health [33]. Though MST is a driving theory in queer mental health disparities research, this theory alone is not enough to inform intervention research to explain queer adolescent suicide risk. MST is valuable in understanding how minority stress impacts queer individuals; however, the original supporting research from which it was developed does not specifically focus on youth (a unique age group), though recently some scholars have applied MST to queer adolescents to address this gap [38-41]. Additionally, MST lacks some depth on important societal contexts (i.e., families, schools, peers, and communities), considerations for transgender youth [42] and other forms of intersectionality, and elaboration about specific suicidality processes and outcomes.

### 4.2. Person-in-Environment and Risk and Resilience (PIE-R&R) Framework

The PIE-R&R framework emphasizes the importance of environmental contexts and systems (e.g., family and schools) in which individuals live and interact to understand human development and psychosocial outcomes [34,43]. For queer youth mental health outcomes, some of the most important social environments include their families, schools and peers, and communities [34,44]. Although these environmental contexts are pertinent for all youth, they play particularly important roles among queer youth who seek acceptance of their identity from family, romantic and sexual partners among peers, and school and community resources to cope with social challenges related to coming out or marginalization stemming from heterosexism or cisgenderism. These social environments have both proximal and distal effects on youth outcomes related to mental health and suicide risk. For example, family members acting in rejecting and abusive ways when a queer youth comes out to them may result in immediate effects of psychological distress and relationship strain. More distally, such negative experiences may manifest into queer youth being in affective states of isolation, shame, and social disconnection, potentially with clinically significant psychological disorders. The work of Fraser and others (e.g., [45-47]) incorporated concepts of risk and protection into the person-in-environment framework to highlight the interplay of factors at the individual and environmental levels that increase or decrease the likelihood of positive and problematic outcomes for people. This work resulted in the PIE-R&R framework [34,42]. *Risk factors* refer to “any event, condition, or experience or that increases the probability that a problem will be formed, maintained, or exacerbated” [48] (p. 5). Conversely, *protective factors* refer to “characteristics, conditions, and resources that buffer or mitigate the impact of risk, interrupt risk processes, or prevent adverse outcomes” [34] (p. 8). Identification of risk and protective factors for suicidality among queer youth can have important implications. Inherent in the PIE-R&R framework are chronological contexts, including developmental factors (e.g., socioemotional development and queer identity development) and sociohistorical context. These factors are highly relevant due to the developmental contexts of queer youth, as the sociocultural dynamics of gender and sexuality can shift and vary across time and place. The PIE-R&R framework underscores the importance of understanding the influences, interactions, and processes of risk and protective factors among individuals and their environments, which has implications for intervention and prevention at multiple levels of the social ecology (e.g., individual, group, community, and systems).

### 4.3. Interpersonal-Psychological Theory of Suicide (IPTS)

IPTS is arguably “the most well-developed theory for suicide” [49] (p. 1) to explain pathways leading individuals to attempt suicide. The main concepts are thwarted belongingness, perceived burdensomeness, acquired capability for suicide [35], and, more recently, *hopelessness* (“one of the most salient states of despair” [50] (p. 2). *Thwarted belongingness* refers to perceived social isolation or detachment, whereas *perceived burdensomeness* relates to an individual’s beliefs that their existence is encumbering to others [35]. These concepts are particularly relevant to queer youth, who may feel ostracized or burdensome to communities and families of origin who may not align with their expansive gender or sexual orientation identities. *Acquired capability for suicide* is a condition marked by fearlessness and pain tolerance toward actions and ideas related to suicide [35]. In addition to feelings of hopelessness, an emotion prevalent among many queer youth [14], IPTS posits that all three concepts overlap, resulting in elevated suicide risk (i.e., suicidal ideation, suicidal intent, suicide attempt, or death [50]. Though heavily tested in suicide research, IPTS as originally proposed does not account for systemic influences such as racism [50], and only recently has it been applied to the study of adolescents in general [51] and queer youth suicide, specifically (e.g., [38,52]. Additionally, although IPTS attempts to explain how suicidal behaviors manifest, less attention is concentrated on “temporal dynamics” (p. 1) of suicide risk, such as when a suicide occurs, or the nonlinear processes that lead to risk [53]. IPTS as a theory alone may miss some important factors (i.e., multi-systemic factors and triggering events) that predict suicide—some individuals attempt suicide without having any known prior suicidal ideation or mental health issues [53,54]. Though some researchers have made efforts to expand upon the IPTS framework to address these limitations (i.e., The Integrated Motivational-Volitional Model of Suicidal Behaviour), empirical support is still growing in this area [55].

### 4.4. Intersectionality Framework

Intersectionality as a conceptual framework was launched into scholarly discourse by Kimberlé Crenshaw in 1989 and has been advanced by other thought leaders (e.g., [56,57]) to illustrate the ways social systems of oppression intersect and shape the lives of individuals and groups with multiple identities and social statuses. Several systems of oppression (e.g., racism, nativism, sexism, cisgenderism, heterosexism, religism, ageism, ableism, and classism) pervade U.S. society and confer advantages to individuals with dominant identities or statuses (e.g., White people, native-born citizens, males, cisgender people, heterosexuals, Christians, adults, people without disabilities/impairments, and wealthy people), while also disadvantaging people with marginalized identities or statuses (e.g., people of color, immigrants, females, transgender people, queer people, non-Christians, children and adolescents, disabled people, and poor or low-income people [58,59]).

Oppression manifests in many ways, including discrimination, violence, exploitation, disempowerment, cultural imperialism, and internalized stigma. Further, a key principle of the Intersectionality framework is that oppressive forces impact individuals differently, depending on one’s set of identities and social statuses, which comprise multiple layers of privilege and/or marginalization, leading to unique intersectional stressors and compounding stress. Despite being crucial in understanding suicide risk, Intersectionality has not been applied ubiquitously nor rigorously in scholarly research with queer youth. Though recently some researchers have advocated for intersectional applications [60,61], historically, adolescent suicide research lacks intersectional components, such as recruiting diverse samples, reporting relevant sociodemographic characteristics (e.g., racial/ethnic identity, gender identity, sexual orientation, disability, socioeconomic status), and incorporating intersectional analyses and interpretations of data in more nuanced and meaningful ways. Youths’ queer identities intersect with many other identities, backgrounds, and social statuses, leading to unique experiences with heterosexism, cisgenderism, and other forms of oppression. These experiences can increase risk for suicide in various ways and must be considered in understanding individual risk and protective factors and creating relevant suicide prevention interventions [7,54].

## 5. An Integrated Conceptual Model to Understand Suicidality among Queer Youth

**Figure A1.**
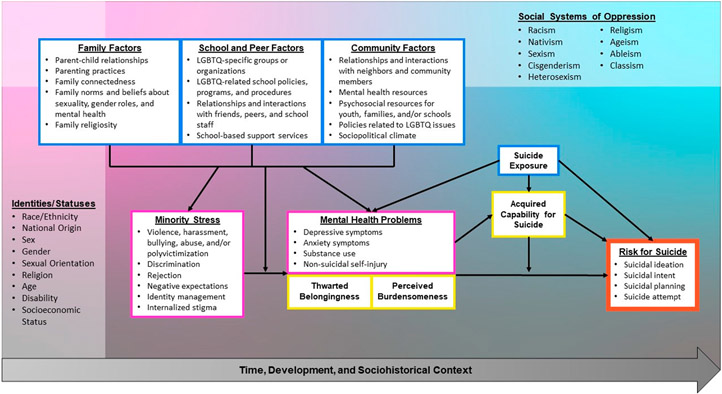
Queer Prevention of Youth Suicidality Model (Queer-PRYSM).

Figure A1 in Appendix A depicts the proposed conceptual model, the Queer Prevention of Youth Suicidality Model (Queer-PRYSM), which integrates components from MST, the PIE-R&R framework, IPTS, and Intersectionality to help explain suicidality among queer youth and to identify targets for prevention and intervention. The colorful gradient background represents Intersectionality and includes social systems of oppression (e.g., racism, cisgenderism, heterosexism) and individual identities (e.g., race/ethnicity, gender, sexual orientation) that converge and overlap in the Queer-PRYSM. These intersections are ubiquitous in the individual lives of queer youth and the social contexts and systems around them and must be considered in understanding the lived experiences of queer youth, risk, and protective factors for suicidality. MST components in the pink boxes show the relationships between *polyvictimization* (i.e., the “co-occurrence of multiple types of victimization” [62] (p. 240), minority stressors specific to queer youth (e.g., internalized stigma), and mental health problems (e.g., depression). The blue boxes represent PIE-R&R proximal social environments (e.g., family, school, peer, and community factors, as well as suicide exposure) that play significant roles in psychosocial processes and mental health outcomes among queer youth related to suicide risk, as supported by theory and extant literature. The family, school and peer, and community factor boxes are touching to represent the overlaps and interactions between these factors. As posited by our model, individual risk for suicide cannot ignore identities and macro-level influences. Outlined in yellow, relationships between IPTS concepts of thwarted belongingness, perceived burdensomeness, and acquired capability for suicide are shown, which can compound the risk for suicide in queer youth. The outcome of risk of suicide (outlined in red) has cognitive and behavioral indicators, including suicidal ideation, suicidal intent, suicidal planning, and suicide attempt. The arrows express relationships between factors and outcomes, including risk and protective factors. An arrow from one factor pointing to another factor or outcome indicates a predictive relationship. An arrow pointing to the axis of another arrow indicates a potentially moderating relationship. These relationships are explored further in the sections below, as supported and indicated by the literature discussed and some case conceptualization application examples based off the research. This holistic, integrated model aims to better explain pathways leading to suicide risk among queer youth, including risk and protective mechanisms, to provide important principles for developing, implementing, and evaluating suicide prevention efforts to ultimately promote mental health equity among queer youth.

### 5.1. Minority Stress

#### 5.1.1. Violence, Polyvictimization, and Abuse

Minority stressors, such as violence, abuse, and rejection, can negatively impact queer youths’ mental well-being and potentially lead to an increase in suicide risk. Queer youth—especially pansexual, bisexual, gender-nonconforming, and/or transgender adolescents—tend to have higher rates of polyvictimization and minority stressors than their heterosexual, cisgender counterparts [62-65]). A growing area of research, some studies have shown links to trauma (e.g., polyvictimization), negative mental health outcomes, and suicide risk in queer youth [62,63].

#### 5.1.2. Discrimination, Rejection, Negative Expectations, and Identity Management

Queer adolescents, especially queer youth of color, are overrepresented in the child welfare system, often due to experiencing violence, abuse, and rejection for their sexual orientation or gender identity by families of origin [66]. In addition to the aforementioned minority stressors, foster care placements that are unwelcoming and hostile to queer youth may contribute to the higher rates of houselessness or rehoming experienced by this population [66]. Such rejecting experiences may increase youths’ internalized stigma and anticipation of negative expectations of others. Queer youth in fear of violence or harassment may conceal their sexual orientation or gender identity, which could translate to mental health problems and risk of suicide due to feelings of rejection or thwarted belongingness.

#### 5.1.3. Internalized Stigma

Queer youth who experience stigma-related stress, family rejection, and multiple forms of bullying or harassment are more likely to report depressive symptoms, distress, and substance use than those who have not faced victimization [67]. Consequently, queer youth minority stress and internalized stigma are associated with lower suicide-related disclosure intentions [68]. Queer youth with higher rates of internalized queer stigma (often broadly referred to in the literature as *“internalized homophobia”*) are more likely to experience suicidal ideation [68], but less likely to report suicidal thoughts [69], especially to adults they feel are unsupportive of their queer identity. Differences in the experience of minority stress and its effects on mental health and suicide risk may also vary among queer youth subgroups depending on their sexual orientations and gender identities. For example, bisexual individuals are more likely than gay, lesbian, or heterosexual youth to experience internalizing symptoms, such as anxiety and depression [20]—mental health outcomes which are positively correlated with internalized biphobia [70]. Among transgender and gender non-conforming youth, research shows that depressive symptoms are associated with internalized transphobia, though this may be moderated by such factors as gender identity appearance, congruence, and resilience [71,72]. Research indicates that minority stress among queer youth (e.g., social marginalization, family rejection, internalized homonegativity, identity management, homonegative climate, negative disclosure experiences and expectancies, homonegative communication) are all significantly associated with mental and behavioral health outcomes [39-41].

### 5.2. Interpersonal and Psychological Factors of Suicide Risk

#### 5.2.1. Mental Health Problems

There are numerous mental health problems facing queer youth that primarily stem from experiences of socially based oppression and can contribute to increased risk for suicide (e.g., depressive disorders, anxiety disorders, substance use disorders, and non-suicidal self-injury). Substantial research shows that queer youth experience high rates of psychological problems, including depression, anxiety, substance use, and non-suicidal self-injury [15,19,73]), which can lead to an increased risk of suicide [74]. Queer youth who feel unsupported in their environment (e.g., family of origin, school, and community) may lack the social supports and coping skills necessary to manage mental health issues, such as depression and anxiety, thereby using maladaptive strategies such as substance use or non-suicidal self-injury to cope. Research shows direct associations between hopelessness, depression, suicidal ideation, and suicide attempts [29,73,75,76]. Some research also shows that there are strong, positive associations between non-suicidal self-injury and suicidality—higher levels of non-suicidal self-injury severity relate to increased suicide risk and negative psychological outcomes [77]. However, queer youth with protective factors in their families, schools, peer groups, and communities, experience less minority stress, and are thereby less likely to experience psychological distress [73] and, subsequently, their risk of suicide may lessen.

Overall, the experience of minority stressors manifests differently among queer youth depending on their various intersectional identities. Per the research cited above, for example, consider the case conceptualization of an individual assigned female at birth, male transgender, bisexual youth in foster care who may otherwise have higher levels of minority stress due to their queer identity. They may be protected by a combination of caring, affirming foster parents, inclusive, supportive school staff and policies, and peer support groups, subsequently lessening their mental health issues and suicide risk. Supportive school climates foster affirming, nonjudgmental spaces, such as those outlined by the Gay, Lesbian & Straight Education Network (GLSEN)’s *Safe Space Kit* [78] can mitigate risk for mental health issues and suicide. Conversely, in this example, a youth with the same background in an environment that is rejecting and hostile toward their queer identity may experience exacerbated mental health issues and suicide risk through victimization, discrimination, and internalized stigma. The queer youth may experience multiple rejections from both the queer community and heterosexual, cisgender communities as someone who identifies as both bisexual and transgender. Negative interactions and continued rejections may lead to an expectation of negative interactions with other, causing them to hide their queer identity (e.g., identity management). If they are unable to feel acceptance and live life as their true, authentic self and experience joy (e.g., gender euphoria), this may decrease the likelihood that they will be able to find supportive individuals in their lives. Such experiences can lead to feelings of thwarted belonging and perceived burdensomeness, as explored more in-depth below.

#### 5.2.2. Thwarted Belongingness and Perceived Burdensomeness

Queer youth who feel ostracized by others because of their sexual orientation and gender identities may feel hopeless and experience alienation by their families, schools, and communities, subsequently increasing their risk for suicide [38,79,80]. For example, per the Queer-PRYSM model, queer youth may disclose their queer identity to their family, and if their parent(s)/guardian(s) do not have a positive reaction, without adequate friend support or supportive social networks to serve as protective factors, they may become suicidal. Youth who feel supported in these areas may experience feelings of hope and acceptance, lessening the likelihood that they will experience suicidal ideation or behaviors. Socio-contextual factors outlined in Queer-PRYSM (e.g., family, school, peer, and community factors) could either positively or negatively impact thwarted belongingness and perceived burdensomeness. Research shows that thwarted belongingness and perceived burdensomeness were significantly positively correlated with severity of suicidal ideation [80], placing queer youth at a higher suicide risk. Moreover, thwarted belongingness, perceived burdensomeness, and acquired capability for suicide are all associated with a history of suicide attempts [80], and individuals with previous suicide attempts are at a higher risk for future attempts [31].

As supported by the literature outlined above, queer youth who are in loving, supportive relationships with adults, community members, and peers may experience less thwarted belonging and less feelings of perceive burdensomeness, but rather, feelings of security and belonging. Research also shows that perceived burdensomeness and non-suicidal self-injury both partially mediated the relationship between depression and suicidal risk [30], consistent with prior research on the link between non-suicidal self-injury and risk of suicide [81], indicating that any relationships that support queer youth and increase their feelings of worthiness may serve as a protective factor against both non-suicidal self-injury and suicide risk. Some studies indicate that sexual minority stress is associated with both perceived burdensomeness and thwarted belongingness in models predicting suicidal ideation and suicide attempt among queer youth [38]. Minority stress has a direct effect on suicide attempt, and an indirect effect on both suicidal ideation and suicide attempt through perceived burdensomeness [38]. In other studies with queer youth, gender identity moderated the relationship between IPTS factors and suicidal ideation [79], and gender identity was significantly associated with perceived burdensomeness, thwarted belongingness, and suicidal ideation.

Congruent with PIE-R&R and MST assumptions and prior research showing that transgender youth have higher risk for suicidal ideation and suicide attempt when compared to cisgender adolescents [28], transgender or gender non-conforming youth reported higher levels of perceived burdensomeness than their cisgender counterparts [79], which may partially explain their heightened levels of suicidal ideation. However, in safe, supportive environments where queer youth receive messages and support affirming sexual orientation and gender, the opposite may be true—being out (i.e., not concealing one’s identity) can translate to better health outcomes [42,76] and a lower risk of suicide.

### 5.3. Socioecological Factors

#### 5.3.1. Family Factors

Family factors can contribute to an individual’s experience of minority stress, dependent upon their identities, with potential to lead to mental health problems and risk of suicide among queer youth through concepts such as perceived burdensomeness and thwarted belongingness. Arguably, for all youth, family relationships are a primary context for defining value and belongingness [49]. Family support is crucial in suicide prevention efforts for queer youth, at times more so than peer support [82]. Most research on parenting practices and adolescent suicide supports the notion that parental connectedness (e.g., through trust, support, validation, and warmth) toward queer youth significantly buffers maladaptive outcomes, such as depression, non-suicidal self-injury (i.e., self-harm), substance use, and suicide risk [49,73,83-87]. Parent connectedness and support for queer youth can be protective in preventing the transition from passive suicidal ideation to suicide attempt [74,82], and queer youth are more likely to talk about suicidal ideation and behaviors with safe, affirming adults [69,88].

In general, positive parent–child relationships are imperative for queer youth well-being and mitigating suicide risk [30,49,81,84]. While warm, structured parenting with clear boundaries can be protective against adolescent suicide attempts, parental rejection can increase risk for suicidality [49]. Navigating a gender and/or sexual minority identity can be difficult, especially for youth living in unaccepting, hostile family environments. Family rejection is linked to adverse mental health outcomes, such as depression and suicidal ideation [83,89], and such rejection may contribute to queer youth houselessness [90]. For queer youth, the process of disclosing their queer status to their family can add to stress, disrupt the family unit, and cause conflict within the parent–child relationship, subsequently elevating feelings of shame, depression [91], and feelings of perceived burdensomeness. This conflict is especially detrimental when family norms and beliefs are entrenched in rigid gender roles (e.g., toxic masculinity, machismo [91]), or religious ideologies that do not support queer relationships [92], which can lead to internalized stigma and suicide risk, as indicated by research with queer young people [68].

Familial homophobic and/or transphobic attitudes or anti-queer sentiments can worsen the internalized stigma of queer youth and lead to negative mental health outcomes [73,91]. If families force queer youth into sexual orientation change efforts (e.g., conversion therapy), research suggests they will be at a higher risk for depression, suicidal ideation, and suicide attempts in their lifetime [93,94]. Feelings of thwarted belongingness may arise in queer youth if subjected to sexual orientation change efforts [93], discrimination [95], and violence [83], depending on their levels and the types of relationships and social support queer youth have in their lives [49].

Some studies show that religious affiliation can also moderate the discrimination-depression relationship among queer youth [92] and lead to positive mental health outcomes if messages on same-sex relations are affirming [95]. Queer youth religious affiliations with denominations that endorse same-sex marriages can mitigate the harmful effects of discrimination and depression symptomology [92,95]. Depressive symptoms are a risk factor for suicidal ideation, non-suicidal self-injury, and suicide attempts among queer youth [83], therefore religious affiliations promoting and supporting sexual minorities can mitigate suicide risk. As such, queer youth family religious affiliations can pose as a risk or protective factor, depending on the group’s beliefs and practices [68,92]. Ongoing peer and adult support are associated with reduced suicide attempts [73], suggesting that the concept of “chosen families,” those who are supportive but may not be related by blood or marriage, could mitigate negative nuclear family interactions and their harmful effects [96].

#### 5.3.2. School and Peer Factors

School and peer factors can contribute to an individual’s experience of minority stress, with potential to either buffer or exacerbate mental health problems and suicide risk in queer youth. Queer youth face similar risk and protective factors as those outlined for all adolescent youth, but their gender and/or sexual minority status makes them more vulnerable to experiencing stress, violence, homelessness, peer bullying, and a lack of support in school [73,83]. Bullying, physical assaults, sexual violence victimization, and arguments with peers regarding queer issues are additional risk factors associated with depression [73], suicidal ideation, and suicidal behaviors [16]. Queer youth, especially bisexual, transgender, and gender non-conforming youth disproportionately experience these issues [97,98], making queer youth more susceptible to suicidal ideation, suicidal behaviors, and death by suicide [83].

Social isolation can involve a limited sense of belonging, an inability to connect with others, and the neglect or deterioration of social relationships [99]. Social isolation can negatively and disproportionately impact queer youth in their family, school, and peer environments [76]. When queer youth experience rejection or bullying, they are at a greater risk of dying by suicide [16]. In one literature review, most studies focused on the experience of social isolation, which often led to non-suicidal self-injury and suicide attempt [99]. Social support levels often predict non-suicidal self-injury—individuals with smaller support networks tend to engage in non-suicidal self-injury more frequently and severely when compared to those with higher support networks [77]. Social systems (e.g., schools) can further promote positive mental health outcomes and reduce suicide risk among queer youth by incorporating policies and programs which promote positive school climate, gender and sexual orientation-related pride, peer support and other forms of adult acceptance (e.g., teachers [44,83,84,100,101].

Positive social school influences and groups for queer youth, such as a Gay-Straight Alliance, Gender and Sexuality Alliance, or Queer-Straight Alliance (GSA/QSA), are associated with significantly lower levels of homophobic and transphobic victimization (e.g., violence, discrimination, rejection) and fear for safety [102]. In a meta-analysis, researchers found ample evidence to support GSAs/QSAs as a means of protecting queer youth from school-based victimization [102]. Given research demonstrating that peer victimization is an antecedent to depressive symptoms [103] and internalizing problems [104], school belonging is a critical protective factor to mitigate minority stress on queer youth mental health and suicide outcomes [84]. Queer youth groups in schools are helpful in reducing suicidal behaviors among all youth, but especially queer youth [105]. Promoting youth connectedness in their school environment and adaptive coping skills [87,106,107] can further promote resiliency among queer youth in schools and communities. Parents and teachers alike can advocate for equal rights of queer youth (e.g., allowing youth to play on sports teams or use bathrooms that align with their gender identity), augmenting their overall well-being [83,108].

While ongoing positive, supportive social connections for gender expansive and queer youth can protect against depression [73], suicidal ideation and suicide risk [83], research also supports the notion that some social connections can augment an individual’s suicide risk through exposure to suicide-related behaviors, or suicide diffusion, as explored in a later section [109,110]. As such, in addition to promoting positive school environments through affirming psychosocial resources, specific supports (e.g., school-based mental health clinicians, suicide prevention and postvention practices) should also be provided for those in crisis to lessen the risk of suicide [111].

#### 5.3.3. Community Factors

Community factors are inherently complex and can subsequently influence the risk of suicide in queer youth. Though not an exhaustive list of community-related factors, relationships between adolescents and others in their community, mental health resources, psychosocial supports, policies, and the sociopolitical climate may serve as protective or risk factors, depending on context [44,112]. Queer youth who have positive interactions with other youth and adults in their communities in terms of affirmation, acceptance, or even respectful tolerance regarding their queer identities would be beneficial to mental health, whereas negative or hostile interactions in community spaces may be harmful. Some communities, such as more progressive urban areas, typically have more queer-specific and queer-affirming mental health treatment and service options. Communities may also vary in opportunities for psychosocial engagement around queer issues, such as queer youth social organizations, support groups for parents and family members of queer youth, and queer youth advocacy organizations that interface with schools. Policies can foster supportive climates for queer youth or stigmatize queer youth, which can shape mental health outcomes; for example, local or state policies may prohibit transgender youth from using bathrooms that align with their identity, whereas other policies prohibit the harmful practices of sexual orientation change efforts with minors [44]. Some evidence suggests that queer youth in urban areas and those in communities with fewer political conservatives may also serve as protective factors, specifically toward depression and suicidality [31,44,73].

Within the larger community context, concealment of identity may present with more positive or negative outcomes, depending on other variables within the individual and their environment [76], such as gender identity, sexual orientation, and government policies [42]. Queer youth with higher levels of outness can seek supportive resources such as support groups that affirm gender and sexual orientation that mitigate minority stress [76,84]. However, outness may put youth at a risk for victimization in the community [83] or discriminatory practices [42], especially in areas where gender and/or sexual minorities are politically oppressed [88]. These stressors, mental health problems, and risk for suicide can change depending on the intersection of various marginalized identities, such as race/ethnicity, disability status, social class, and age [36].

#### 5.3.4. Suicide Exposure and Acquired Capability for Suicide

Suicide exposure and acquired capability for suicide are combined here and stacked in our model as we suggest they are somewhat related, but suicide exposure relates more to PIE-R&R, whereas the originates from IPTS. In general, adolescents are more vulnerable to suicide after exposure to a suicide loss or a suicide attempt in their social groups [109,110,113]. Referred to as *suicide contagion* by some scholars, these authors prefer the term *suicide diffusion* to indicate an exposure to a peer’s suicide, which can result in suicidal ideation or suicidal behaviors in adolescents and last for years, especially when combined with other risk factors [107,114]. Queer youth have higher rates of suicide attempts and deaths when compared to their heterosexual and cisgender counterparts [12]. When exposed to sensationalized media reports of suicide or the trauma of a suicide death or attempt in one’s social circles, queer youth with pre-existing mental health conditions and other risk factors are likely to experience heightened levels of suicidal ideation and/or risk of a suicide attempt themselves [107]. Considering high rates of internet use among queer youth [115], exposure to sensationalized, detailed articles, stories, or forums related to suicide attempt, death, and non-suicidal self-injurious behaviors may further contribute to the risk of suicide [116]. Additionally, suicide loss survivors or people who lose significant individuals in their lives to suicide are at an elevated threat for suicide [117], complicated grief [118], and other mental health problems [119,120], placing queer youth with a potential for exacerbated suicide risk. However, it is important to note that the internet can also serve as a protective factor by way of queer youth being able to connect with one another, and locate resources that may not be accessible in their locality (e.g., suicide bereavement online forums, mental health crises hotline numbers).

Suicide exposure and subsequent risk of suicide death in queer youth suicide attempt and suicide bereaved survivors may be related through an individual’s acquired capability for suicide, as they may become less fearful of pain and death through each subsequent attempt or exposure. However, in addition to disagreements on the definitions, “transmission”, and influence of suicide diffusion among youth [121], there are other unknowns and uncertainties on the relationships between suicide loss and exposure. Some studies indicate that quality and closeness of relationships with the deceased (i.e., individual who died by suicide) are associated with the effects of grief and mental health issues, though other studies find there are no differences in number of suicide attempts among suicide-exposed adolescents versus those who have not been exposed to a suicide death [122]. Social supports and their effects among suicide-bereaved adolescents, mental health, their views on suicide, and help-seeking also have mixed findings depending on various relationship dynamics [122]. Our model reflects these relationships through the connection between family, school, peer, and community factors, as they relate to exposure to suicide-related behaviors and the acquired capability for suicide. Queer youth who experience a suicide loss in their lives, especially among a close family member or friend, are at a greater risk of developing mental health problems such as depression, anxiety, substance use, non-suicidal self-injury. These issues could be compounded by experiences of minority stress, subsequently increasing their risk of suicide. However, protective factors such as safe, queer-affirming relationships and other community factors such as mental health supports may mitigate the risk of suicide, especially when combined with universal interventions such as postvention in and outside of schools [18,117].

In addition to exposure to suicide and/or suicide-related behaviors, the relationships between non-suicidal self-injury and suicide risk might be better understood through the concept of acquired capability for suicide [35]. As queer youth engage in non-suicidal self-injury, they may become more pain tolerant and less fearful of suicide, which may explain correlations between non-suicidal self-injury, suicide attempt, and death [31]. Acquired capability for suicide may also partially explain the relationship between history of suicide attempt and suicide death [31], as well as the link between accessibility to lethal means (e.g., guns) and its connection to completed suicides, especially among youth in rural settings [123]. Queer youth in rural settings may be particularly susceptible to this suicide risk when considering the school, peer, and community factors indicated in Queer-PRYSM if they are isolated and unable to find affirming, supportive individuals in their lives and due to a dearth of mental health resources.

### 5.4. Intersectionality: Identities and Systems of Oppression

Social systems of oppression create a challenging environment for queer youth. In addition to anti-queer bias, other systems of oppression (e.g., racism, sexism, classism) overlap and jointly shape the context of queer youth suicide risk (e.g., [22]). The disproportionately high levels of suicidal ideation and suicidal behaviors among the queer youth population [14,16,19] can be explained by examining the points of intersecting identities (e.g., queer youth of color, disabled queer youth). The systems of oppression experienced at intersecting identities (e.g., sexual orientation and race, gender identity and disability) influence other aspects in the youth’s life (e.g., family and community factors), and possibly, the differential outcomes of queer subgroup risk and protective factors. For instance, queer youth of color can experience high rates of racist/heterosexist/cisgenderist bullying and discrimination unique to them due to the intersecting oppressive systems confronting them. Furthermore, they face exclusion based on race/ethnicity in mainstream White queer spaces [124] and may also experience less family acceptance of their queer identity [125]. Disabled queer youth also have been shown to have high rates of suicide risk [21], highlighting the need to examine identity points with high social adversity. All together, these experiences thwart social belonging with family, peers, and communities, while leading to unique forms of minority stress and mental health problems, such as anxiety and depression [126]. Queer, non-binary or transgender youth of color may experience additional difficulties in accessing affirming care and mental health treatment due to systemic racism, cisgenderism, heterosexism, and other barriers based on both their identity and related social systems of oppression. Queer-PRYSM can be used to explore unique manifestations of risk factors (e.g., discrimination and thwarted belongingness) for queer youths’ intersecting identities that stem from oppressive systems as well as unique protective factors (e.g., community relationships and interactions). Varying social contexts have implications for the responses to youth suicide risk such as appropriate treatment targets and the leveraging of youth’s resources and strengths.

## 6. Discussion

Theories and conceptual models help researchers, practitioners, policymakers, and others conceptualize, explain, study, and address complex psychosocial and health problems, with an ultimate goal to advance social justice and health equity. This article presented a new conceptual model that integrated elements from four established theories or concepttual frameworks and their associated evidence. Queer-PRYSM has implications for suicide research, intervention, and prevention.

### 6.1. Implications for Research and Policy

Etiological research is needed to identify substantial factors that increase risk for suicide among queer youth. Although Queer-PRYSM presents various risk factors, some likely exert a stronger influence than others in terms of distress leading to suicidality. For example, experiencing violence or rejection may be more severe events contributing to risk for suicidality compared to having negative expectations, which may be a less severe form of minority stress. In addition, extant and new policies that fail to protect queer youth or are explicitly hostile to queer youth (e.g., school anti-bullying laws, policies restricting medical care for transgender youth, “Don’t Say Gay or Trans” policies [127] may be risk factors for suicidality. Research on risk pathways and mechanisms can also identify mediators that may be important targets of intervention to prevent the escalation of suicide risk (e.g., acquired capability for suicide).

Researchers should also investigate moderating factors that could mitigate risk (i.e., protective factors). The literature on suicide risk and environmental influences disproportionately focuses on risk factors over protective factors [31]. In terms of protection, having queer-specific school supports could moderate the effects of minority stressors on psychological outcomes (e.g., depression, lack of belonging). Similarly, certain factors may prevent risk for suicidality among queer youth. Having largely respectful, tolerant, and/or affirming interactions in one’s family, school, and/or peer group may prevent queer youth from ever becoming at risk for suicidality. Queer-PRYSM’s inclusion of systems and environmental contexts along with more proximal individual and interpersonal factors necessitates the examination of how factors from different socioecological levels interact and shape suicidality outcomes. Queer-PRYSM’s emphasis on intersectionality indicates a need for research on how factors related to intersecting identities increase or decrease youth’s risk for suicide, as well as identifying various pathways in oppression-related stress, distress, and suicidality for subgroups of queer youth with particular identities. In addition to broad, general intervention strategies (i.e., gatekeeper trainings, postvention), more targeted interventions are likely needed for particular groups of queer youth who have differing experiences that increase risk for suicide. Queer-PRYSM also has implications for intervention and prevention research.

### 6.2. Implications for Practice

Given the many stakeholders, systems, phases, and levels involved in the suicidality of queer youth as depicted in Queer-PRYSM, there are multiple targets for prevention and intervention. Etiological research could inform the development of assessment tools or systems to measure key indicators or risk factors for suicidality, as well as strengths and resources that could be leveraged to mitigate suicide risk. Assessment of suicide risk could identify queer youth needing intervention to prevent the escalation of psychological problems and suicidality. As indicated by our model, suicide prevention efforts should not only focus on individual interventions, but on the broader, macro-level as well. Queer-PRYSM suggests several avenues for prevention and intervention regarding queer youth suicidality that span the public health social work intervention continuum, which includes universal prevention, selective intervention, and treatment and direct services [34]. For example, universal prevention could occur by having a sufficient number of mental health professionals (e.g., school counselors and social workers) available at schools to address student issues; though these human service resources are not specifically intended for queer youth, these students could benefit from such mental health resources, which may prevent suicidality among queer youth. In addition to this, staff must be trained in basic knowledge related to queer youth, such as the importance of gender-affirming language, pronouns, and issues specifically facing their queer students in order to best support them (e.g., [128]). Continuing with schools as an example, there may be specific school infrastructure for queer students to prevent suicidality, given that queer youth are a group at increased risk for suicide. This could include a GSA/QSA, which is a type of student-based organization in middle schools or high schools of queer and allied students that engage in community building, education, advocacy, and/or social support around gender and sexuality issues. The presence of a GSA/QSA, though not specifically focused on suicide prevention, may help decrease risk for suicidality among queer youth by providing social support to queer youth and creating a more affirming school climate [129]. For queer youth who are struggling with psychosocial or mental health problems (e.g., thwarted belonging and depression) that are strong indicators of heightened risk for suicidality, more targeted interventions are necessary. Such interventions may include group counseling or psychotherapy to target distress from queer-specific stressors (e.g., shame regarding identity, lack of affirming community, coping with discrimination). Finally, youth exhibiting any form of suicidality (e.g., suicidal ideation and behavior) require direct services to prevent suicide. Queer youth in this difficult psychological space may need multiple interventions, including safety planning, mental health treatment, and cultivating affirming interpersonal relationships.

## 7. Conclusions

The proposed conceptual model integrated relevant elements from four broader theories/frameworks, and extant empirical literature was described and cited as available. Explicating a model with comprehensive explanatory capacity, modifiable psychosocial factors, and directional and interacting pathways yields a myriad of promising opportunities in assessing needs, promoting areas of strength, and identifying targeted areas of intervention to address the societal and public health concern of suicidality among queer youth, with the goal of saving lives.

## References

[1] Ertl A; Crosby AE; Blair JM Youth suicide: An opportunity for prevention. J. Am. Acad. Child Adolesc. Psychiatry 2020, 59, 1019–1021. 32861417 10.1016/j.jaac.2020.01.017 PMC7510279

[2] Hawkins JD; Jenson JM; Catalano R; Fraser MW; Botvin GJ; Shapiro V; Hendricks Brown C; Beardslee W; Brent D; Leslie LK; Unleashing the Power of Prevention; National Academy of Medicine: Washington, DC, USA, 2015.

[3] Centers for Disease Control & Prevention. Preventing Suicide. 2021. Available online: https://www.cdc.gov/suicide/pdf/preventing-suicide-factsheet-2021-508.pdf (accessed on 23 May 2022).

[4] Elfein J Distribution of the 10 Leading Causes of Death among Teenagers Aged 15 to 19 Years in the United States in 2019. Statista, 2021. Available online: https://www.statista.com/statistics/1017959/distribution-of-the-10-leading-causes-of-death-among-teenagers/ (accessed on 23 May 2022).

[5] Ramchand R; Gordon JA; Pearson JL Trends in suicide rates by race and ethnicity in the United States. JAMA Netw. Open 2021, 4, e2111563. 34037735 10.1001/jamanetworkopen.2021.11563 PMC8155821

[6] Kohlbeck S; Hargarten S; Cassidy LD Age- and sex-specific risk factors for youth suicide: A mixed methods review. Wis. Med. J 2020, 119, 165–170. 33091283

[7] Alvarez K; Polanco-Roman L; Samuel Breslow A; Molock S Structural racism and suicide prevention for ethnoracially minoritized youth: A conceptual framework and illustration across systems. Am. J. Psychiatry 2022, 179, 422–433. 35599542 10.1176/appi.ajp.21101001 PMC9765395

[8] Braudt DB; Lawrence EM; Tilstra AM; Rogers RG; Hummer RA Family socioeconomic status and early life mortality risk in the United States. Matern. Child Health J 2019, 23, 1382–1391. 31273497 10.1007/s10995-019-02799-0 PMC6732231

[9] Gordon J Addressing the Crisis of Black Youth Suicide; National Institute of Mental Health: Bethesda, MD, USA, 2020. Available online: https://www.nimh.nih.gov/about/director/messages/2020/addressing-the-crisis-of-black-youth-suicide (accessed on 28 February 2022).

[10] Yildiz M; Orak U; Walker MH; Solakoglu O Suicide contagion, gender, and suicide attempts among adolescents. Death Stud. 2019, 43, 365–371. 29920166 10.1080/07481187.2018.1478914

[11] Conron KJ LGBT Youth Population in the United States; University of California, Los Angeles Williams Institute School of Law: Los Angeles, CA, USA, 2020. Available online: https://williamsinstitute.law.ucla.edu/wp-content/uploads/LGBT-Youth-US-Pop-Sep-2020.pdf (accessed on 23 February 2022).

[12] Adelson SL; Graeme R; Miller AM; Sandfort TGM Health justice for LGBT youths: Combining public health and human rights. J. Am. Acad. Child Adolesc. Psychiatry 2021, 60, 804–807. 33711378 10.1016/j.jaac.2021.02.021

[13] Toomey RB; Syvertsen AK; Shramko M Transgender adolescent suicide behavior. Pediatrics 2018, 142, e20174218. 30206149 10.1542/peds.2017-4218 PMC6317573

[14] Centers for Disease Control and Prevention. 1991–2019 High School Youth Risk Behavior Survey Data. 2019. Available online: http://nccd.cdc.gov/youthonline (accessed on 18 August 2022).

[15] Johns MM; Lowry R; Andrzejewski J; Barrios LC; Demissie Z; Rasberry CN; Robin L; Underwood JM Transgender identity and experiences of violence victimization, substance use, suicide risk, and sexual risk behaviors among high school students—19 States and Large Urban School Districts, 2017. Morb. Mortal. Wkly. Rep 2019, 68, 67–71. 10.15585/mmwr.mm6803a3 PMC6348759 30677012

[16] Horwitz AG; Grupp-Phelan J; Brent D; Barney BJ; Casper TC; Berona J; Chernick LS; Shenoi R; Cwik M; King CA Risk and protective factors for suicide among sexual minority youth seeking emergency medical services. J. Affect. Disord 2021, 279, 274–281. 33074147 10.1016/j.jad.2020.10.015 PMC7738357

[17] Nock MK; Borges G; Bromet EJ; Cha CB; Kessler RC; Lee S Suicide and suicidal behavior. Epidemiol. Rev 2008, 30, 133–154. 18653727 10.1093/epirev/mxn002 PMC2576496

[18] Singer JB; Erbacher TA; Rosen P School-based suicide prevention: A framework for evidence-based practice. School Ment. Health 2019, 11, 54–71.

[19] The Trevor Project. National Survey on LGBTQ Youth Mental Health 2021. 2021. Available online: https://www.thetrevorproject.org/survey-2021/?section=SuicideMentalHealth (accessed on 28 February 2022).

[20] Borgogna NC; McDermott RC; Aita SL; Kridel MM Anxiety and depression across gender and sexual minorities: Implications for transgender, gender nonconforming, pansexual, demisexual, asexual, queer, and questioning individuals. Psychol. Sex. Orientat. Gend. Divers 2019, 6, 54–63.

[21] Tejera CH; Horner-Johnson W; Andresen EM Application of an intersectional framework to understanding the association of disability and sexual orientation with suicidal ideation among Oregon teens. Disabil. Health J 2019, 12, 557–563. 31167741 10.1016/j.dhjo.2019.05.006

[22] Dawes HC; Williams DY; Klein LB; Hirst LE; Forte AB; Gibbs DJ; Hall WJ Experiences of LGBTQ POC in mental health services and substance abuse: A systematic review. J. Soc. Soc. Work Res 2022.

[23] Hughes E; Rawlings V; McDermott E Mental health staff perceptions and practice regarding self-harm, suicidality and help-seeking in LGBTQ youth: Findings from a cross-sectional survey in the UK. Issues Ment. Health Nurs 2018, 39, 30–36. 29369735 10.1080/01612840.2017.1398284

[24] Galster G; Sharkey P Spatial foundations of inequality: A conceptual model and empirical overview. Russell Sage Found. J. Soc. Sci 2017, 3, 1–33. Available online: https://www.jstor.org/stable/10.7758/rsf.2017.3.2.01 (accessed on 6 June 2022).

[25] Barth RP; Messing JT; Shanks TR; Williams JH (Eds.) Grand Challenges for Social Work and Society, 2nd ed.; Oxford University Press: New York, NY, USA, 2022.

[26] Hatchel T; Polanin JR; Espelage DL Suicidal thoughts and behaviors among LGBTQ youth: Meta-analyses and a systematic review. Arch. Suicide Res 2021, 25, 1–37. 31597541 10.1080/13811118.2019.1663329

[27] Price-Feeney M; Green AE; Dorison S Understanding the mental health of transgender and nonbinary youth. J. Adolesc. Health 2020, 66, 684–690. 31992489 10.1016/j.jadohealth.2019.11.314

[28] Thoma BC; Salk RH; Choukas-Bradley S; Goldstein TR; Levine MD; Marshal MP Suicidality disparities between transgender and cisgender adolescents. Pediatrics 2019, 144, e20191183. 31611339 10.1542/peds.2019-1183 PMC7011156

[29] Hirsch JK; Cohn TK; Rowe CA; Rimmer SE Minority sexual orientation, gender identity status and suicidal behavior: Serial indirect effects of hope, hopelessness and depressive symptoms. Int. J. Ment. Health Addict 2017, 15, 260–270.

[30] Kang N; You J; Huang J; Ren Y; Lin M-P; Xu S Understanding the pathways from depression to suicidal risk from the perspective of the interpersonal–psychological theory of suicide. Suicide Life-Threat. Behav 2018, 49, 684–694. 29578277 10.1111/sltb.12455

[31] Franklin JC; Ribeiro JD; Fox KR; Bentley KH; Kleiman EM; Huang X; Musacchio KM; Jaroszewski AC; Chang BP; Nock MK Risk factors for suicidal thoughts and behaviors: A meta-analysis of 50 years of research. Psychol. Bull 2017, 143, 187–232. 27841450 10.1037/bul0000084

[32] Meyer IH Minority stress and mental health in gay men. J. Health Soc. Behav 1995, 36, 38–56. 7738327

[33] Meyer IH Prejudice, social stress, and mental health in lesbian, gay, and bisexual populations: Conceptual issues and research evidence. Psychol. Bull 2003, 129, 674–697. 12956539 10.1037/0033-2909.129.5.674 PMC2072932

[34] Hall WJ; Lanier P; Jenson JM; Fraser MW A multisystems risk and resilience approach to social policy for children, youth, and families. In Social Policy for Children and Families: A Risk and Resilience Perspective, 4th ed.; Hall WJ, Lanier P, Jenson JM, Fraser MW, Eds.; Sage: New York, NY, USA, 2022; pp. 1–26.

[35] Joiner TE Why People Die by Suicide; Harvard University Press: Cambridge, MA, USA, 2005.

[36] Crenshaw K Mapping the margins: Intersectionality, identity politics, and violence against women of color. Stanf. Law Rev 1990, 43, 1241.

[37] Hendricks ML; Testa RJ A conceptual framework for clinical work with transgender and gender nonconforming clients: An adaptation of the Minority Stress Model. Prof. Psychol. Res. Pract 2012, 43, 460.

[38] Fulginiti A; Goldbach JT; Mamey MR; Rusow J; Srivastava A; Rhoades H; Schrager SM; Bond DW; Marshal MP Integrating minority stress theory and the interpersonal theory of suicide among sexual minority youth who engage crisis services. Suicide Life-Threat. Behav 2020, 50, 601–616. 32048340 10.1111/sltb.12623

[39] Goldbach JT; Schrager SM; Mamey MR Criterion and divergent validity of the Sexual Minority Adolescent Stress Inventory. Front. Psychol 2017, 8, 2057. 29234292 10.3389/fpsyg.2017.02057 PMC5712417

[40] Goldbach JT; Schrager SM; Mamey MR; Rhoades H Confirming the reliability and validity of the Sexual Minority Adolescent Stress Inventory in a national sample of sexual minority adolescents. Front. Psychol 2021, 12, 720199. 34531800 10.3389/fpsyg.2021.720199 PMC8438190

[41] Goldbach JT; Gibbs JJ A developmentally informed adaptation of minority stress for sexual minority adolescents. J. Adolesc 2017, 55, 36–50. 28033502 10.1016/j.adolescence.2016.12.007 PMC5272836

[42] Hoy-Ellis CP Minority stress and mental health: A review of the literature. J. Homosex 2021, 1–25. 10.1080/00918369.2021.2004794 34812698

[43] Kondrat ME Person-in-environment. In Oxford Research Encyclopedia of Social Work; Franklin C, Ed.; National Association of Social Workers Press: Washington, DC, USA; Oxford University Press: Oxford, UK, 2013.

[44] Hatzenbuehler ML The social environment and suicide attempts in lesbian, gay, and bisexual youth. Pediatrics 2011, 127, 896–903. 21502225 10.1542/peds.2010-3020 PMC3081186

[45] Fraser MW The ecology of childhood: A multisystems perspective. In Risk and Resilience in Childhood, 2nd ed.; Fraser MW, Ed.; NASW Press: Washington, DC, USA, 2004; pp. 1–12.

[46] Fraser MW; Richman JM; Galinsky MJ Risk, protection, and resilience: Towards a conceptual framework for social work practice. Soc. Work Res 1999, 23, 131–144.

[47] Fraser MW; Kirby LD; Smokowski PR Risk and resilience in childhood. In Risk and Resilience in Childhood: An Ecological Perspective, 2nd ed.; Fraser MW, Ed.; NASW Press: Washington, DC, USA, 2004; pp. 13–66.

[48] Fraser MW; Terzian MA Risk and resilience in child development: Practice principles and strategies. In Handbook of Children, Youth, and Family Services: Practice, Policies, and Programs; Mallon GP, Hess PM, Eds.; Columbia University Press: New York, NY, USA, 2005; pp. 55–71.

[49] Diamond G; Kodish T; Ewing ES K; Hunt QA; Russon JM Family processes: Risk, protective and treatment factors for youth at risk for suicide. Aggress. Violent Behav 2022, 64, 101586. 35662798 10.1016/j.avb.2021.101586 PMC9159634

[50] Joiner TE; Jeon ME; Lieberman A; Janakiraman A; Duffy ME; Gai AR; Dougherty SP On prediction, refutation, and explanatory reach: A consideration of the interpersonal theory of suicidal behavior. Prev. Med 2021, 152, 106453. 34538380 10.1016/j.ypmed.2021.106453

[51] Calear AL; McCallum S; Kazan D; Werner-Seidler A; Christensen H; Batterham PJ Application of the interpersonal psychological theory of suicide in a non-clinical community-based adolescent population. J. Affect. Disord 2021, 294, 235–240. 34303302 10.1016/j.jad.2021.07.011

[52] Grossman AH; Park JY; Russell ST Transgender youth and suicidal behaviors: Applying the interpersonal psychological theory of suicide. J. Gay Lesbian Ment. Health 2016, 20, 329–349. 28344728 10.1080/19359705.2016.1207581 PMC5363722

[53] Bryan CJ; Butner JE; May AM; Rugo KF; Harris J; Oakey DN; Rozek DC; Bryan AO Nonlinear change processes and the emergence of suicidal behavior: A conceptual model based on the fluid vulnerability theory of suicide. New Ideas Psychol. 2021, 57, 1–22. 10.1016/j.newideapsych.2019.100758 PMC7050543 32123464

[54] Buchmann-Schmitt JM; Chiurliza B; Chu C; Michaels MS; Joiner TE Suicidality in adolescent populations: A review of the extant literature through the lens of the interpersonal theory of suicide. Int. J. Behav. Consult. Ther 2014, 9, 26–34.

[55] O’Connor RC; Kirtley OJ The integrated motivational-volitional model of suicidal behaviour. Philos. Trans. R. Soc. B 2018, 373, 20170268. 10.1098/rstb.2017.0268 PMC6053985 30012735

[56] Bowleg L The problem with the phrase women and minorities: Intersectionality—An important theoretical framework for public health. Am. J. Public Health 2012, 102, 1267–1273. 22594719 10.2105/AJPH.2012.300750 PMC3477987

[57] Collins PH Intersectionality’s definitional dilemmas. Ann. Rev. Sociol 2015, 41, 1–20.

[58] Garcia B; Van Soest D Oppression. In Encyclopedia of Social Work; Franklin C, Ed.; National Association of Social Workers Press: Washington, DC, USA; Oxford University Press: Oxford, UK, 2019.

[59] Young IM Five faces of oppression. In Readings for Diversity and Social Justice, 4th ed.; Adams M, Blumenfeld WJ, Catalano DCJ, DeJong KS, Hackman HW, Hopkins LE, Love BJ, Peters ML, Shlasko D, Zuniga X, Eds.; Routledge: London, UK, 2018; pp. 49–58.

[60] Shangani S; Gamarel KE; Ogunbajo A; Cai J; Operario D Intersectional minority stress disparities among sexual minority adults in the USA: The role of race/ethnicity and socioeconomic status. Cult. Health Sex 2020, 22, 398–412. 31144598 10.1080/13691058.2019.1604994 PMC6884660

[61] Williams SL; Job SA; Todd E; Braun K A critical deconstructed quantitative analysis: Sexual and gender minority stress through an intersectional lens. J. Soc. Issues 2020, 76, 859–879.

[62] Johns MM; Lowry R; Hipp TN; Robin L; Shafir S Differences in adolescent experiences of polyvictimization and suicide risk by sexual minority status. J. Res. Adolesc 2021, 31, 240–252. 33232570 10.1111/jora.12595 PMC8048776

[63] Green AE; Price-Feeney MN; Dorison SH Cumulative minority stress and suicide risk among LGBTQ youth. Am. J. Community Psychol 2022, 69, 157–168. 34534356 10.1002/ajcp.12553

[64] Kassing F; Casanova T; Griffin JA; Wood E; Stepleman LM The effects of polyvictimization on mental and physical health outcomes in an LGBTQ sample. J. Trauma. Stress 2021, 34, 161–171. 33269807 10.1002/jts.22579

[65] Sterzing PR; Gartner RE; Goldbach JT; McGeough BL; Ratliff GA; Johnson KC Polyvictimization prevalence rates for sexual and gender minority adolescents: Breaking down the silos of victimization research. Psychol. Violence 2019, 9, 419–430.

[66] Interagency Working Group on Youth Programs. Child Welfare. In Youth.Gov.; 2015. Available online: https://youth.gov/youth-topics/lgbtq-youth/child-welfare#_ftn (accessed on 10 February 2022).

[67] Scheer JR; Edwards KM; Helminen EC; Watson RJ Victimization typologies among a large national sample of sexual and gender minority adolescents. LGBT Health 2021, 8, 507–518. 34619055 10.1089/lgbt.2021.0024 PMC9022181

[68] Gibbs JJ; Goldbach J Religious conflict, sexual identity, and suicidal behaviors among LGBT young adults. Arch. Suicide Res 2015, 19, 472–488. 25763926 10.1080/13811118.2015.1004476 PMC4706071

[69] Chang CJ; Kellerman J; Feinstein BA; Selby EA; Goldbach JT Greater minority stress is associated with lower intentions to disclose suicidal thoughts among LGBTQ plus youth. Arch. Suicide Res 2022, 26, 626–640. 32970971 10.1080/13811118.2020.1818656

[70] Dyar C; Feinstein BA; Sarno EL; Pirog S; Newcomb ME; Whitton SW Prospective associations between bi+ minority stressors and internalizing symptoms: The mediating roles of general and group-specific processes. J. Consult. Clin. Psychol 2021, 89, 845–855. 34807659 10.1037/ccp0000689 PMC8725783

[71] Chodzen G; Hidalgo MA; Chen D; Garofalo R Minority stress factors associated with depression and anxiety among transgender and gender-nonconforming youth. J. Adolesc. Health 2019, 64, 467–471. 30241721 10.1016/j.jadohealth.2018.07.006 PMC6528476

[72] Jackman KB; Dolezal C; Bockting WO Generational differences in internalized transnegativity and psychological distress among feminine spectrum transgender people. LGBT Health 2018, 5, 54–60. 29099335 10.1089/lgbt.2017.0034 PMC5770087

[73] Hall WJ Psychosocial risk and protective factors for depression among lesbian, gay, bisexual, and queer youth: A systematic review. J. Homosex 2018, 65, 263–316. 28394718 10.1080/00918369.2017.1317467 PMC5634914

[74] Taliaferro LA; Muehlenkamp JJ Risk and protective factors that distinguish adolescents who attempt suicide from those who only consider suicide in the past year. Suicide Life-Threat. Behav. 2014, 44, 6–22. 23855367 10.1111/sltb.12046

[75] Rogers ML; Joiner TE Rumination, suicidal ideation, and suicide attempts: A meta-analytic review. Rev. Gen. Psychol 2017, 21, 132–142.

[76] Timmins L; Rimes KA; Rahman Q Minority stressors, rumination, and psychological distress in lesbian, gay, and bisexual individuals. Arch. Sex. Behav 2020, 49, 661–680. 31332645 10.1007/s10508-019-01502-2 PMC7031186

[77] O’Loughlin CM; Gomer B; Ammerman BA The social context of nonsuicidal self-injury: Links to severity, suicide risk, and social factors. J. Clin. Psychol 2021, 77, 1004–1017. 33084062 10.1002/jclp.23073

[78] Gay, Lesbian & Straight Education Network. The Safe Space Kit: Guide to Being an Ally to LGBT Students. 2016. Available online: https://www.glsen.org/sites/default/files/GLSEN%20Safe%20Space%20Kit.pdf (accessed on 19 October 2022).

[79] Chang CJ; Feinstein BA; Fulginiti A; Dyar C; Selby EA; Goldbach JT A longitudinal examination of the interpersonal theory of suicide for predicting suicidal ideation among LGBTQ+ youth who utilize crisis services: The moderating effect of gender. Suicide Life-Threat. Behav 2021, 51, 1015–1025. 34156125 10.1111/sltb.12787 PMC8551000

[80] Chu C; Buchman-Schmitt JM; Stanley IH; Hom MA; Tucker RP; Hagan CR; Rogers ML; Podlogar MC; Chiurliza B; Ringer FB; The interpersonal theory of suicide: A systematic review and meta-analysis of a decade of cross-national research. Psychol. Bull 2017, 143, 1313–1345. 29072480 10.1037/bul0000123 PMC5730496

[81] Almeida J; Johnson RM; Corliss HL; Molnar BE; Azrael D Emotional distress among LGBT youth: The influence of perceived discrimination based on sexual orientation. J. Youth Adolesc 2009, 38, 1001–1014. 19636742 10.1007/s10964-009-9397-9 PMC3707280

[82] Mustanski B; Liu RTA Longitudinal study of predictors of suicide attempts among lesbian, gay, bisexual, and transgender youth. Arch. Sex. Behav 2013, 42, 437–448. 23054258 10.1007/s10508-012-0013-9

[83] Gorse M Risk and protective factors to LGBTQ+ youth suicide: A review of the literature. Child Adolesc. Soc. Work J 2020, 39, 17–28.

[84] Katz-Wise SL; Sarda V; Austin SB; Harris SK Longitudinal effects of gender minority stressors on substance use and related risk and protective factors among gender minority adolescents. PLoS ONE 2021, 16, e0250500. 34077452 10.1371/journal.pone.0250500 PMC8171963

[85] Kidd S; Henrich CC; Brookmeyer KA; Davidson L; King RA; Shahar G The social context of adolescent suicide attempts: Interactive effects of parent, peer, and school social relations. Suicide Life-Threat. Behav 2006, 36, 386–395. 16978093 10.1521/suli.2006.36.4.386

[86] Ryan C; Russell ST; Huebner D; Diaz R; Sanchez J Family acceptance in adolescence and the health of LGBT young adults. J. Child Adolesc. Psychiatr. Nurs 2010, 23, 205–213. 21073595 10.1111/j.1744-6171.2010.00246.x

[87] Whitlock J; Wyman PA; Moore SR Connectedness and suicide prevention in adolescents: Pathways and implications. Suicide Life-Threat. Behav 2014, 44, 246–272. 24444252 10.1111/sltb.12071

[88] Puckett JA; Horne SG; Surace F; Carter A; Noffsinger-Frazier N; Shulman J; Detrie P; Ervin A; Mosher C Predictors of sexual minority youth’s reported suicide attempts and mental health. J. Homosex 2017, 64, 697–715. 27268386 10.1080/00918369.2016.1196999

[89] Schmitz RM; Robinson BA; Sanchez J Intersectional family systems approach: LGBTQ+ Latino/a youth, family dynamics, and stressors. Fam. Relat 2020, 69, 832–848.

[90] McKay T; Berzofsky M; Landwehr J; Hsieh P; Smith A Suicide etiology in youth: Differences and similarities by sexual and gender minority status. Child. Youth Serv. Rev 2019, 102, 79–90.

[91] Mereish EH; Cox DJ; Harris JC; Anderson QR Emerging ideas: Familial influences, shame, guilt, and depression among sexual minority adolescents. Fam. Relat 2021, 70, 1546–1555.

[92] Gattis MN; Woodford MR; Han Y Discrimination and depressive symptoms among sexual minority youth: Is gay-affirming religious affiliation a protective factor? Arch. Sex. Behav 2014, 43, 1589–1599. 25119387 10.1007/s10508-014-0342-y PMC4507415

[93] Blosnich JR; Henderson ER; Coulter RWS; Goldbach JT; Meyer IH Sexual orientation change efforts, adverse childhood experiences, and suicide ideation and attempt among sexual minority adults, United States, 2016–2018. Am. J. Public Health 2020, 110, 1024–1030. 10.2105/AJPH.2020.305637 PMC7287530 32437277

[94] Ryan C; Toomey RB; Diaz RM; Russell ST Parent-initiated sexual orientation change efforts with LGBT adolescents: Implications for young adult mental health and adjustment. J. Homosex 2018, 67, 159–173. 30403564 10.1080/00918369.2018.1538407 PMC10371222

[95] Anderson OS; McGuire JK “I feel like God doesn’t like me”: Faith and ambiguous loss among transgender youth. Fam. Relat 2021, 70, 390–401.

[96] Green AE; Price-Feeney M; Dorison SH Association of sexual orientation acceptance with reduced suicide attempts among lesbian, gay, bisexual, transgender, queer, and questioning youth. LGBT Health 2020, 8, 26–31. 33275858 10.1089/lgbt.2020.0248

[97] Caputi TL; Shover CL; Watson RJ Physical and sexual violence among gay, lesbian, bisexual, and questioning adolescents. JAMA Pediatr. 2020, 174, 791–793. 32150233 10.1001/jamapediatrics.2019.6291 PMC7063536

[98] Gordon AR; Conron KJ; Calzo JP; White MT; Reisner SL; Austin SB Gender expression, violence, and bullying victimization: Findings from probability samples of high school students in 4 U.S. school districts. J. School Health 2018, 88, 306–314. 29498058 10.1111/josh.12606 PMC5836796

[99] Garcia J; Vargas N; Clark JL; Magaña Álvarez M; Nelons DA; Parker RG Social isolation and connectedness as determinants of well-being: Global evidence mapping focused on LGBTQ youth. Glob. Public Health 2020, 15, 497–519. 31658001 10.1080/17441692.2019.1682028 PMC7093214

[100] Day JK; Fish JN; Grossman AH; Russell ST Gay-straight alliances, inclusive policy, and school climate: LGBTQ youths’ experiences of social support and bullying. J. Res. Adolesc 2020, 30, 418–430. 30861243 10.1111/jora.12487 PMC8063225

[101] Kosciw JG; Palmer NA; Kull RM; Greytak EA The effect of negative school climate on academic outcomes for LGBT youth and the role of in-school supports. J. School Violence 2013, 12, 45–63.

[102] Marx RA; Kettrey HH Gay-straight alliances are associated with lower levels of school-based victimization of LGBTQ+ youth: A systematic review and meta-analysis. J. Youth Adolesc 2016, 45, 1269–1282. 27221632 10.1007/s10964-016-0501-7

[103] Hatchel T; Espelage DL; Huang Y Sexual harassment victimization, school belonging, and depressive symptoms among LGBTQ adolescents: Temporal insights. Am. J. Orthopsychiatr 2018, 88, 422–430. 10.1037/ort0000279 28617003

[104] Kiekens W; La Roi C; Bos HM W; Kretschmer T; Van Bergen DD; Veenstra R Explaining health disparities between heterosexual and LGB adolescents by integrating the minority stress and psychological mediation frameworks: Findings from the TRAILS study. J. Youth Adolesc 2020, 49, 1767–1782. 32076922 10.1007/s10964-020-01206-0 PMC7423798

[105] Poteat VP; Sinclair KO; DiGiovanni CD; Koenig BW; Russell ST Gay-straight alliances are associated with student health: A multischool comparison of LGBTQ and heterosexual youth. J. Res. Adolesc 2013, 23, 319–330.

[106] Marraccini ME; Brier ZM School connectedness and suicidal thoughts and behaviors: A systematic meta-analysis. School Psychol. Q 2017, 32, 5. 10.1037/spq0000192 PMC5359058 28080099

[107] Poland S; Lieberman R; Niznik M Suicide Contagion and Clusters—Part 1: What School Psychologists Should Know. Communiqué 2019, 47, 21–23. Available online: https://www.nasponline.org/publications/periodicals/communique/issues/volume-47-issue-5/suicide-contagion-and-clusters%E2%80%94part-1-what-school-psychologists-should-know (accessed on 23 March 2020).

[108] Davies RD; Kessel B Gender minority stress, depression, and anxiety in a transgender high school student. Am. J. Psychiatry 2017, 174, 1151–1152. 29191036 10.1176/appi.ajp.2017.17040439

[109] Abrutyn S; Mueller AS Are suicidal behaviors contagious in adolescence? Using longitudinal data to examine suicide suggestion. Am. Sociol. Rev 2014, 79, 211–227. 26069341 10.1177/0003122413519445 PMC4461080

[110] Abrutyn S; Mueller AS; Osborne M Rekeying cultural scripts for youth suicide: How social networks facilitate suicide diffusion and suicide clusters following exposure to suicide. Soc. Ment. Health 2020, 10, 112–135.

[111] Poland S; Ferguson S Youth suicide in the school context. Aggress. Violent Behav 2021, 16, 101579.

[112] Fontanella CA; Hiance-Steelesmith DL; Phillips GS; Bridge JA; Lester N; Sweeney HA; Campo JV Widening rural-urban disparities in youth suicides, United States, 1996–2010. JAMA Pediatr. 2015, 169, 466–473. 25751611 10.1001/jamapediatrics.2014.3561 PMC4551430

[113] Randall JR; Nickel NC; Colman I Contagion from peer suicidal behavior in a representative sample of American adolescents. J. Affect. Disord 2015, 186, 219–225. 26253902 10.1016/j.jad.2015.07.001

[114] O’Neill JC Are Schools Prepared for Suicide Contagion Effects? An Analysis of School Psychologists’ Perceive Competency in Postvention Response. (UMI No. 10791912). Ph.D. Thesis, University of North Carolina at Chapel Hill, Chapel Hill, NC, USA, 2017.

[115] Siegel M Everyone Needs Broadband Access—Especially LGBTQ Youth; New America: Washington, DC, USA, 2021; Available online: https://www.newamerica.org/oti/blog/everyone-needs-broadband-access-especially-lgbtq-youth/ (accessed on 10 March 2022).

[116] Marchant A; Hawton K; Stewart A; Montgomery P; Singaravelu V; Lloyd K; Purdy N; Daine K; John A A systematic review of the relationship between internet use, self-harm and suicidal behaviour in young people: The good, the bad and the unknown. PLoS ONE 2017, 12, e0181722. 28813437 10.1371/journal.pone.0181722 PMC5558917

[117] Andriessen K Can postvention be prevention? Crisis 2009, 30, 43–47. 19261568 10.1027/0227-5910.30.1.43

[118] Levi-Belz Y; Lev-Ari L “Let’s talk about it”: The moderating role of self-disclosure on complicated grief over time among suicide survivors. Int. J. Environ. Res. Public Health 2019, 16, 3740. 31590225 10.3390/ijerph16193740 PMC6801618

[119] Cerel J; Campbell FR Suicide survivors seeking mental health services: A preliminary examination of the role of an active postvention model. Suicide Life-Threat. Behav 2008, 38, 30–34. 18355106 10.1521/suli.2008.38.1.30

[120] Cha JM; Kim JE; Kim MA; Shim B; Cha MJ; Lee JJ; Han DH; Chung US Five months follow-up study of school-based crisis intervention for Korean high school students who experienced a peer suicide. J. Korean Med. Sci 2018, 33, e192. 29983694 10.3346/jkms.2018.33.e192 PMC6033103

[121] Cheng Q; Li H; Silenzio V; Caine ED Suicide contagion: A systematic review of definitions and research utility. PLoS ONE 2014, 9, e108724. 25259604 10.1371/journal.pone.0108724 PMC4178222

[122] Andriessen K; Draper B; Dudley M; Mitchell PB Bereavement after suicide: Disentangling clues to better help bereaved adolescents. Crisis 2015, 36, 299–303. 26502780 10.1027/0227-5910/a000339

[123] Capps RE; Michael KD; Jameson JP Lethal means and adolescent suicidal risk: An expansion of the PEACE Protocol. J. Rural Ment. Health 2019, 43, 3–16.

[124] Ghabrial MA “Trying to figure out where we belong”: Narratives of racialized sexual minorities on community, identity, discrimination, and health. Sex. Res. Soc. Policy 2017, 14, 42–55.

[125] Hart TA; Sharvendiran R; Chikermane V; Kidwai A; Grace D At the intersection of homophobia and racism: Sociocultural context and the sexual health of South Asian Canadian gay and bisexual men. Stigma Health 2021.

[126] Thoma BC; Huebner DM Health consequences of racist and antigay discrimination for multiple minority adolescents. Cult. Divers. Ethn. Minor. Psychol 2013, 19, 404–413. 10.1037/a0031739 PMC4086429 23731232

[127] Movement Advancement Project. LGBTQ Youth Issues. 2022. Available online: https://www.lgbtmap.org/ (accessed on 1 March 2022).

[128] GLSEN. Research and Education Webinars. Available online: https://www.glsen.org/resources/webinars-and-workshops (accessed on 9 September 2022).

[129] Fetner T; Elafros A The GSA difference: LGBTQ and ally experiences in high schools with and without gay-straight alliances. Soc. Sci 2015, 4, 563–581.

